# Diurnal and seasonal variability of CO_2_ and CH_4_ concentration in a semi-urban environment of western India

**DOI:** 10.1038/s41598-021-82321-1

**Published:** 2021-02-03

**Authors:** Abirlal Metya, Amey Datye, Supriyo Chakraborty, Yogesh K. Tiwari, Dipankar Sarma, Abhijit Bora, Nirmali Gogoi

**Affiliations:** 1 Indian Institute of Tropical Meteorology, MoES, Pune, 411008 India; 2 Department of Atmospheric and Space Sciences, Savitribai Phule Pune University, Pune, 411007 India; 3 Department of Environmental Science, Tezpur Central University, Tezpur, India

**Keywords:** Biogeochemistry, Environmental sciences

## Abstract

Amongst all the anthropogenically produced greenhouse gases (GHGs), carbon dioxide (CO_2_) and methane (CH_4_) are the most important, owing to their maximum contribution to the net radiative forcing of the Earth. India is undergoing rapid economic development, where fossil fuel emissions have increased drastically in the last three decades. Apart from the anthropogenic activities, the GHGs dynamics in India are governed by the biospheric process and monsoon circulation; however, these aspects are not well addressed yet. Towards this, we have measured CO_2_ and CH_4_ concentration at Sinhagad, located on the Western Ghats in peninsular India. The average concentrations of CO_2_ and CH_4_ observed during the study period are 406.05 ± 6.36 and 1.97 ± 0.07 ppm (µ ± 1σ), respectively. They also exhibit significant seasonal variabilities at this site. CH_4_ (CO_2_) attains its minimum concentration during monsoon (post-monsoon), whereas CO_2_ (CH_4_) reaches its maximum concentration during pre-monsoon (post-monsoon). CO_2_ poses significant diurnal variations in monsoon and post-monsoon. However, CH_4_ exhibits a dual-peak like pattern in pre-monsoon. The study suggests that the GHG dynamics in the western region of India are significantly influenced by monsoon circulation, especially during the summer season.

## Introduction

Carbon dioxide (CO_2_) is one of the minor (~ 0.4% of all gaseous species) constituents of the atmosphere. Still, it plays the most significant role in the radiation balance of the planet among the species produced anthropogenically. CO_2_ contributes 73% of all positive radiative forcing of the Earth's environment since the pre-industrial era, circa 1750s^1,2^. CO_2_ is continuously being exchanged between the terrestrial biosphere, ocean, and the atmosphere^3^ and maintained a more or less steady-state, until about 1750. The balance in CO_2_ is being perturbed due to the anthropogenic emission of CO_2_ and its feedback with the global climate change since the industrialisation^4^. The atmospheric concentration of CO_2_ has progressively increased since the beginning of the industrialisation, from 280 ppm (in 1700) to a current level of more than 410 ppm. About one-fourth of the CO_2_ emissions from the anthropogenic activities (fossil-fuel consumption, cement production, and land cover land-use changes) have been absorbed by the ocean and another one fourth by the terrestrial biosphere during the 2000s^1,5^. The exchange of carbon among the various reservoirs is controlled by complex biogeochemical processes and takes place on various timescales^6^. A better understanding of the carbon exchange process, especially on a short temporal and spatial timescale, is necessary to have a better estimate of the carbon budget^7,8^. This requires a robust network of continuous monitoring of CO_2_ concentration and determining its fluxes from different reservoirs^4,9–11^.

Methane (CH_4_) is the second-largest contributor, among the anthropogenically produced species, to global warming with a positive radiative forcing of about 0.48 ± 0.05 W m^−2^^2^. The pre-industrial CH_4_ concentration was estimated to be 700 ppb, but increased anthropogenic activities have resulted in a steady increase of atmospheric CH_4_, up to 1803 ppb in 2011^12,13^. Apart from being a potent greenhouse gas, CH_4_ plays an active role in tropospheric chemistry. CH_4_ is the main contributor to the increase in stratospheric water vapour, following the loss by reaction with OH radical^14^. The water vapour variation in the upper troposphere and lower stratosphere is highly significant due to its impact on global warming. CH_4_ emissions from anthropogenic sources in India have increased from 18.85 to 20.56 Tg year^−1^ from 1985 to 2008^15^. Unlike CO_2_, methane has a relatively short lifetime of approximately 10 years^16^. Thus, in comparison with CO_2_, CH_4_ can attain a steady-state condition and start to decrease reasonably fast if emissions are stabilised or reduced. However, increased emission of CH_4_, mainly due to human activities, could perturb the equilibrium state. The source and sink mechanism of CH_4_ is complex, and their pathways remain poorly constrained. Apart from a few mid-to-upper tropospheric observations of CH_4_ by satellite remote sensing, its surface monitoring over the Indian subcontinent is sparse. Hence, the key drivers for its diurnal or seasonal scale variability are not well understood. The seasonal variation of CH_4_ over different parts of India can be attributed to the complex interaction between surface emissions and convective transport during monsoon as well as monsoon circulation^17,18^. For example, the eastern Himalayan station Darjeeling captures episodes of higher CH_4_ concentrations throughout the year^19^. A north-western Himalayan station Hanle experiences high values during the summer monsoon season, while Pondicherry, located on the eastern coast of India, and Port Blair, situated on an island in the Bay of Bengal, show comparatively lower values^18^. During June–September, CH_4_ maxima at Hanle is likely due to enhanced biogenic emission from wetlands and rice paddies. Also, deep convection associated with monsoon mixes surface emission to mid-to-upper troposphere enhances the CH_4_ concentration at Hanle. Moreover, the elevated CH_4_ is also found in 8–12 km over a vast region 50°–80° E and south of 40° N during the CARIBIC (Civil Aircraft for the Regular Investigation of the Atmosphere Based on an Instrument Container) aircraft observations^20^. Minimum CH_4_ at Pondicherry and Port Blair during the monsoon season is associated with transportation of southern hemispheric CH_4_-depleted air at low altitudes and high rates of OH oxidation^18^. The large scale observational network is required for understanding the spatial and temporal variations in CH_4_ over India.

India is one of the largest and fastest-growing economies in South Asia and is a significant contributor to CO_2_ emissions in this region. Observations of diurnal and seasonal variations in the contribution of anthropogenic and biogenic sources of CO_2_ and CH_4_ are well documented from many urban stations in Europe and the USA, but only a few cases are documented in the Asian region^21–23^. Tiwari et al.^24^ have shown that CO_2_ variability during winter months (seasonal amplitude) is higher (approx. double) than the summer months at a surface observational site in India. The monitoring was based on flask samples at the weekly interval, but no data is available yet at higher temporal resolution. The short-term variations, such as on diurnal scale, capture the signature of the photosynthesis process, respiration, and anthropogenic emission on the observed variability of atmospheric CO_2_. Hence, it is interesting to identify the time-varying characteristics of CO_2_ and CH_4_ concentrations and the probable causes, which characterise the variability in different time scales over peninsular India.

In view of the above, continuous measurement of CO_2_ and CH_4_ have been carried out from July-2014 to November-2015 from Sinhagad, a Western Ghat site, using a highly sensitive laser-based technique. These measurements are utilised for studying the temporal variations (diurnal and seasonal) of both the gases and identifying the key drivers of such variations, especially the effect of monsoon circulation. The effects of meteorology as well as vegetation dynamics on CO_2_ and CH_4_ concentration on different time scales are also investigated.

## Study area

**Figure 1:**
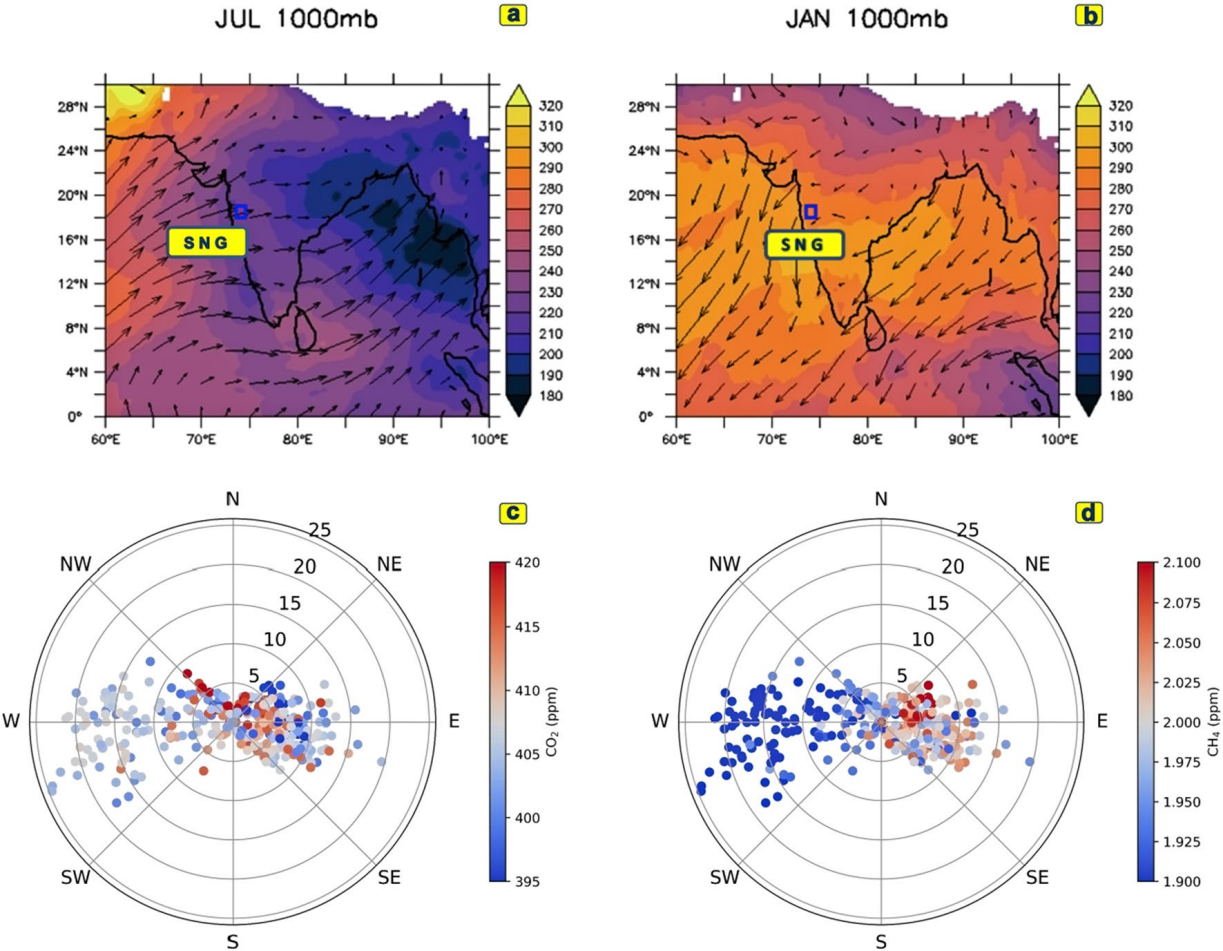
The outgoing longwave radiation on a monthly scale (shaded) at the surface (1000 mb) during (**a**) July (average of 2014–2015) and (**b**) January (2015). Arrows indicate wind vectors. A blue rectangle marks station Sinhagad. The wind rose diagram for (**c**) CO_2_ and (**d**) CH_4_ are also shown.

The study area (Sinhagad; denoted as *sng:* 18° 21′ N, 73° 45′ E, 1600 m above msl) is a semi-urban location in the Western Ghats, India. This region is positioned at a distance of 30 km south-west from the city of Pune and 200 km east from the coastline of the Arabian Sea in Maharashtra, India. The basic climatology is presented in Fig. 1. The outgoing longwave radiations (OLR), on a monthly scale for the summer and winter seasons, are shown in Fig. 1a,b, respectively. The corresponding circulations are depicted by arrows. The windrose diagrams for CO_2_ and CH_4_ are also shown (Fig. 1c,d).

## Results

### Seasonal variation of CO_2_ and CH_4_

**Figure 2:**
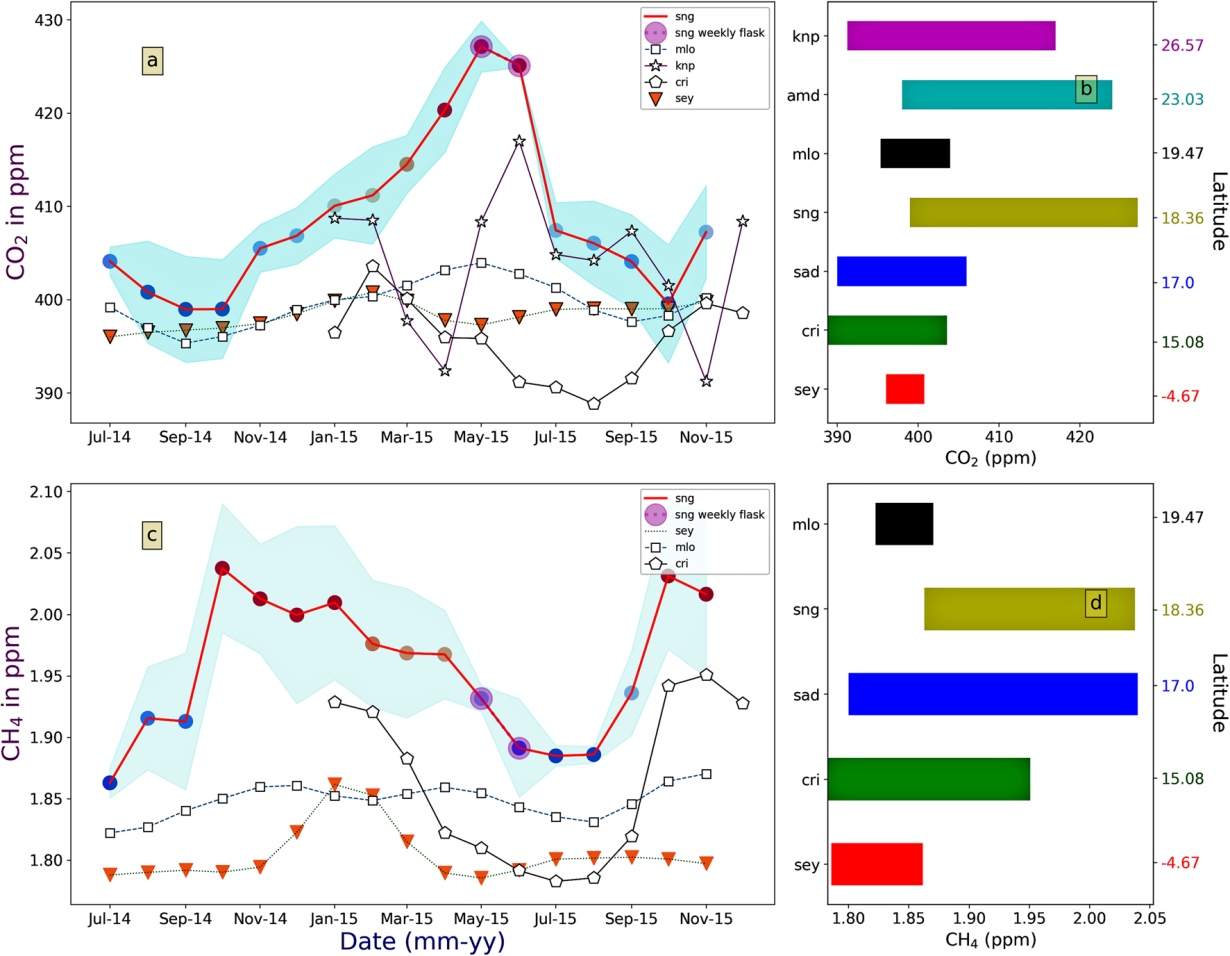
Seasonal variation of (**a**) CO_2_ and (**c**) CH_4_ at Sinhagad, for the year 2014–2015. The shaded region shows the standard deviation. Seasonal variations of other sites are also shown in the figure. *N.B-Seasonal variation of knp is obtained in the year 2016, but it is shown in 2015 in the graph for comparison purpose only. Similarly, cri seasonality*^33^* is obtained from monthly mean data of 2010–2012 but shown in 2015 in the graph for comparison only. Highlighted markers in sng time series in May and June, 2015 is obtained from weekly flask samples to fill the gap.* Seasonal amplitude (maximum − minimum) of several sites is shown in (**b**) for CO_2_ and in (**d**) for CH_4_.

Figure 2a,c shows the monthly mean and standard deviation (SD; shaded region) of CO_2_ and CH_4_ concentrations, respectively. The annual mean concentration of CO_2_ is 406.05 ± 6.36 (µ ± 1σ) ppm. CO_2_ is maximum (427.2) in May-2015 and a minimum (399) in September-2014. This leads to a seasonal amplitude of ~ 28 ppm. A comparison of the seasonal amplitude of other sites, global (*Seychelles*-sey, *Mauna Loa*-mlo), and Indian sites (*Kaziranga*-knp, *Ahmedabad*-amd, *Shadnagar*-sad, *Cabo de Rama*-cri), are shown in Fig. 2b. *sey* and *mlo* data are taken from ESRL-NOAA, *knp* data is obtained under Metflux India^25^ project. *amd* and *sad* seasonality are taken from Chandra et al.^22^ and Sreenivas et al.^26^, *cri* data is taken from World Data Centre for Greenhouse Gases (WDCGG). The global sites *sey* and *mlo* are mostly oceanic, hence possess smaller seasonal variation. In contrast, *knp* is a forest site of north-east India. It shows a larger seasonality of ~ 25 ppm, with a minimum during pre-monsoon and post-monsoon (Fig. 2a). Ahmedabad is an urban site in western India and has a CO_2_ seasonality of 26 ppm. Shadnagar is a semi-urban site in central India with seasonality of 16 ppm. *cri* is a coastal region on the west coast of India. The mean seasonal amplitude of *cri* is 20 ppm with a minimum in monsoon and maximum in February–March. Among all these sites, *sng* shows maximum seasonal amplitude with pre-monsoon maximum and post-monsoon minima. Mean values of CO_2_ for different seasons are 403.34 ± 5.71, 402.87 ± 6.03, 409.72 ± 4.33, 417.06 ± 5.11 ppm during the monsoon, post-monsoon, winter, and pre-monsoon, respectively. Mean CO_2_ increases about 6.85 ppm from post-monsoon to winter and again increases about 7.34 ppm from winter to pre-monsoon.

The annual mean value of CH_4_ over the study region is 1.97 ± 0.07 (µ ± 1σ) ppm. CH_4_ concentration is minimum (1.863 ppm) in July-2014 and maximum (2.037 ppm) in October-2014 (Fig. 2c). The seasonal pattern over *cri* is very similar to the *sng*. The *sng* CH_4_ shows 2.29 times and 3.62 times more seasonality than global sites *sey* and *mlo* (Fig. 2d). Whereas *sad* shows more seasonal amplitude of ~ 240 ppb than *sng* (~ 174 ppb). While *cri* seasonal amplitude, 168 ppb, is very close to *sng* seasonal amplitude, 174 ppb. The average values of CH_4_ in different seasons are 1.903 ± 0.0412, 2.024 ± 0.0567, 1.995 ± 0.0629, 1.966 ± 0.0466 ppm in monsoon, post-monsoon, winter, and pre-monsoon, respectively.

#### Influence of large scale circulation

**Figure 3:**
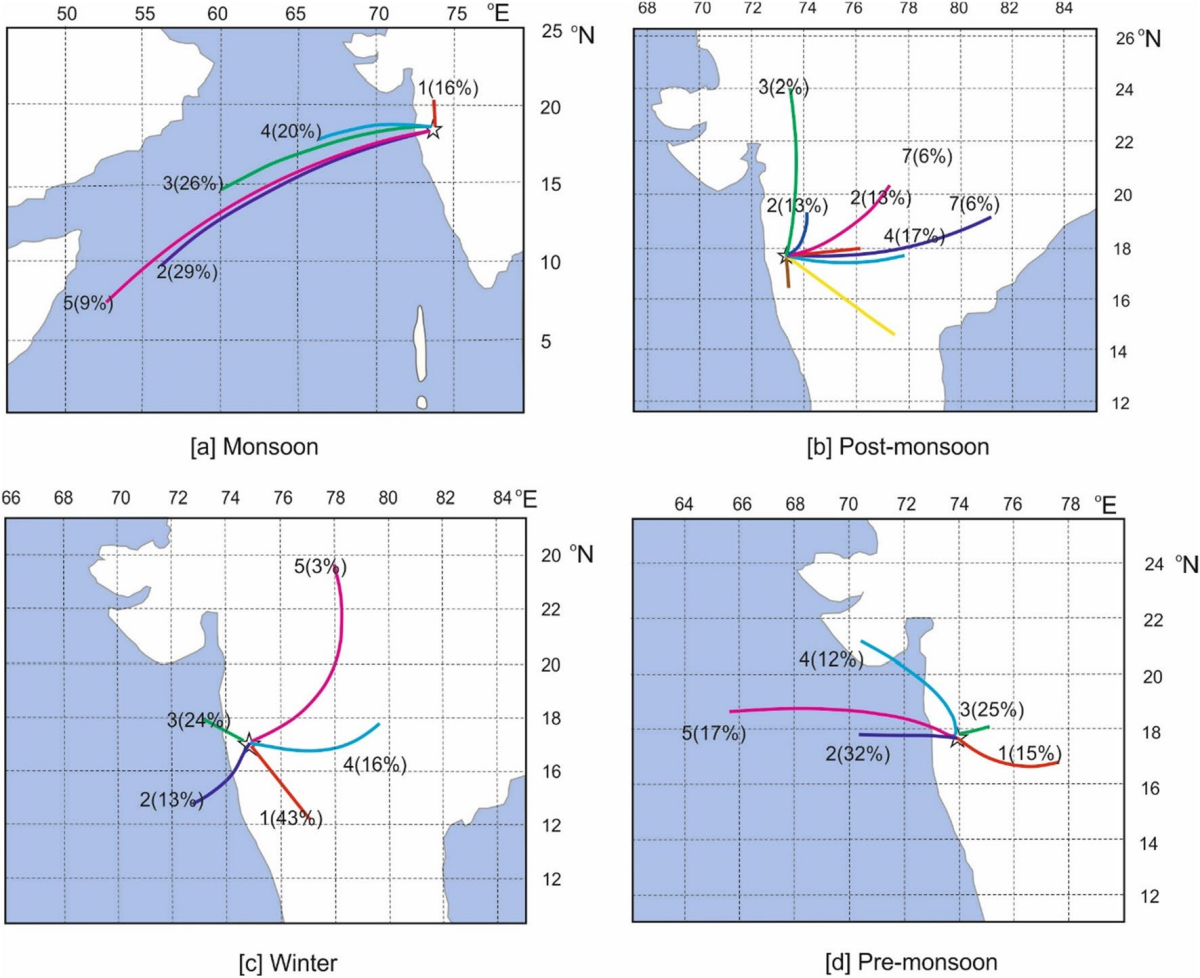
(**a**–**d**) 10-day back trajectories arriving at Sinhagad at a surface level during monsoon, post-monsoon, winter, and pre-monsoon.

To understand the large-scale meteorological circulation, we have used Hybrid Single-Particle Lagrangian Integrated Trajectory model (HYSPLIT), developed by NOAA’s Air Resources Laboratory. We computed 10-day back-trajectory starting from *sng* from June-2014 to November-2015 using NCEP/NCAR reanalysis dataset. The reanalysis data is available from the year 1948 up to present-time with 6-h temporal resolution and 2.5° × 2.5° spatial resolution. The dataset is produced jointly by the National Center for Environmental Prediction (NCEP) and the National Center for Atmospheric Research (NCAR). Trajectories were created in each 6-h interval from *sng*. Then we separated the trajectories into clusters for separate seasons. These clusters are mean trajectories of the air mass. Their percentage contribution to the total, calculated for different seasons over the study period at surface level, is presented in Fig. 3a–d.

Figure 3a reveals that *sng* receives almost 84% of wind from the Arabian sea due to south-west monsoon flow. During the post-monsoon season, the wind blows from the Indian sub-continent. Therefore, the post-monsoon wind carries the contaminated air from the continental region to the *sng* site. During pre-monsoon time, *sng* receives 50% air mass from the Arabian Sea and 50% from the Indian continent. So, the observed maximum CO_2_ concentration during pre-monsoon may be a local phenomenon, not a large scale transport.

#### Influence of vegetation

**Figure 4:**
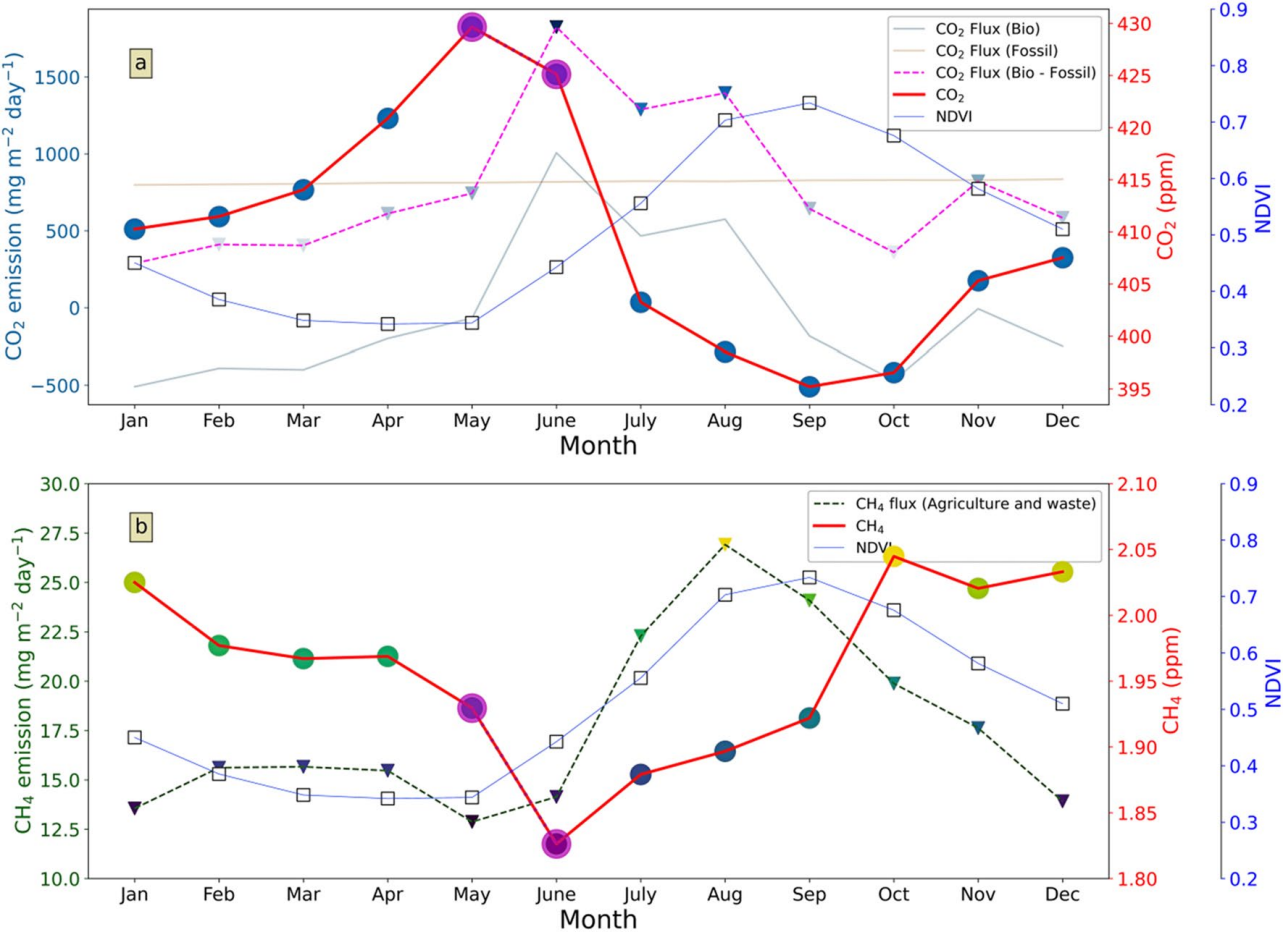
(**a**) Co-variance of CO_2_ and NDVI calculated over an area of 0.5° × 0.5° for the entire observation period. (**b**) Co-variation of CH_4_, NDVI, and CH_4_ flux from agriculture and waste of CT-product. *N.B-*The highlighted points in CO_2_ and CH_4_ time series denotes data from weekly flask samples.

The normalised difference vegetation index (NDVI) is widely used as an index of vegetation cover of a given region^27, 28^. We have plotted CO_2_ and CH_4_ as well as NDVI to investigate their relationship. The monthly climatology of CO_2_, CH_4,_ and NDVI are shown in Fig. 4a,b. Additionally, monthly climatology (2000–2010) of sector-wise CH_4_ emission from Carbon-Tracker (CT) is plotted in Supplementary Fig. 3. It is quite clear that agriculture and waste management practices are the dominant sector of CH_4_ over the study region. Hence, the monthly-climatology of CH_4_ emission from agriculture and waste is also shown in Fig. 4b. Moreover, fossil fuel and biospheric emission of CO_2_ and their residual is also plotted in Fig. 4a.

An inverse correlation is found between CO_2_ and NDVI (Fig. 4a). NDVI time series reveals the growth of vegetation starts from the monsoon. Also, the growth rate is higher during the monsoon season than the non-monsoon season. The NDVI data clearly shows an enhanced vegetation cover from August and a concurrent decrease of CO_2_ in our study region. Increased vegetation cover increases the rate of photosynthesis, which helps in decreasing CO_2_ concentration. Further, NDVI reduces from post-monsoon to winter and pre-monsoon months, and CO_2_ concentration consequently rises. This result is also supported by Sreenivas et al.^26^, who found a negative correlation between NDVI and CO_2_ concentration at *sad* for 2014. Moreover, residual flux (biosphere + fossil) is high positive (positive sign denotes CO_2_ added to the atmosphere) during June–July–August, though atmospheric CO_2_ concentration is low (Fig. 4a).

CH_4_ emission from the agriculture and waste (AW) sector of CT consists of enteric fermentation, animal waste management, wastewater and landfills, and rice agriculture. Emission from the AW sector is high during monsoon. The co-occurrence of high NDVI and AW sector emission suggest that rice agriculture is a dominant part of AW sector emission. In comparison, low surface CH_4_ concentration is observed in monsoon.

#### Influence of planetary boundary layer (PBL)

The planetary boundary layer (PBL) is the lowermost layer of the troposphere, where temperature and wind speed plays an essential role in its height variation. The boundary layer can mix the GHG emitted at the ground level up to a certain height and reduce its concentration near the ground. So, seasonal changes in the boundary layer may affect the ground concentration of GHGs. Monthly PBLH is computed by averaging the hourly data and compared with CO_2_ and CH_4_ concentrations. Monthly PBLH is observed to be minimum (maximum) during the monsoon (pre-monsoon). Seasonal PBLH during monsoon, post-monsoon, winter, and pre-monsoon is 754.8, 1136.45, 1213.72, and 1420.08 m, respectively. The influence of PBLH on CO_2_ and CH_4_ is shown in Supplementary Fig. 2a,b, respectively, for the seasonal transitions, i.e., monsoon to post-monsoon (M-PM), post-monsoon to winter (PM-W), and winter to pre-monsoon (W-PreM). Here ΔPBLH and ΔGHG’s are calculated from the (later season value − previous season value), i.e., ΔPBLH for M-PM means ([PBLH during post-monsoon] − [PBLH during monsoon]).

We find two cases:

1. [ΔPBLH_M-PM_ > ΔPBLH_PM-W_] leads to [ΔCO_2 M-PM_ < ΔCO_2 PM-W_] and [ΔCH_4 M-PM_ > ΔCH_4 PM-W_ ]
2. [ΔPBLH_W-PreM_ > ΔPBLH_PM-W_] leads to [ΔCO_2 W-PreM_ > ΔCO_2 PM-W_] and [ΔCH_4 W-PreM_ < ΔCH_4 PM-W_ ]

### Effect of meteorology in different seasons

**Figure 5:**
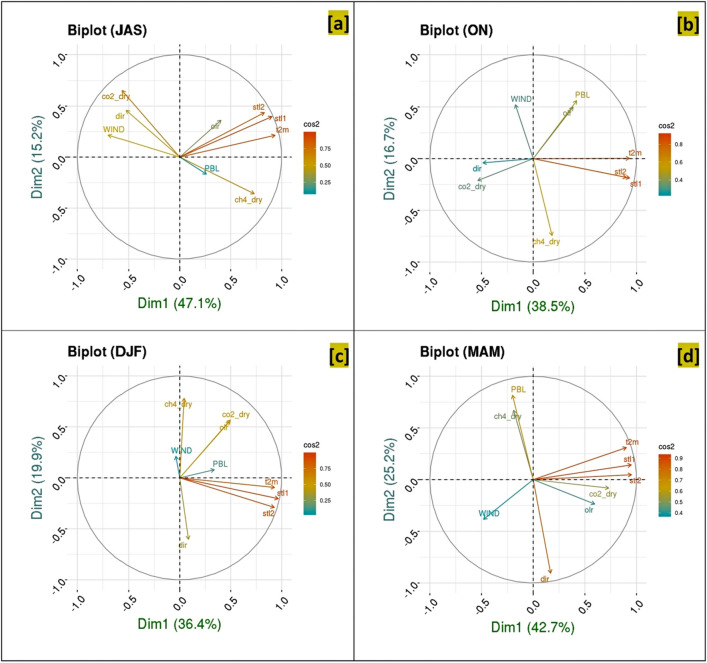
Biplot in PC1 and PC2 space showing the association of individual variables and their phase relationship for (**a**) monsoon (July–August–September), (**b**) post-monsoon (October–November), (**c**) winter (December–January–February), and (**d**) pre-monsoon (March–April–May).

A biplot analysis is carried out for each season to identify the interdependency of several meteorological parameters such as wind speed (WIND), wind direction (dir), outgoing longwave radiation (olr), planetary boundary layer (PBL), 2 m-air temperature (t2m), soil temperature in layer 0–7 cm (stl1) and soil temperature in layer 7–28 cm (stl2) with GHGs (Fig. 5a–d). In the two-dimensional space of two leading principal components, we used the biplot technique^29^ to describe the PCA result. The two axes in the biplot represent the first two principal components, and the arrow vectors describe the variables in this space. Supplementary Fig. 8a–d shows the scree plot for monsoon, post-monsoon, winter, and pre-monsoon, respectively. Scree plot is the plot of eigenvalues organised from largest to smallest. Here scree plot is shown in terms of the percentage of explained variance. It is to be noted that in each season, the first two PCs (PC1 and PC2) are dominant components; hence, biplot analysis is carried out for each season to identify the interdependency of several meteorological parameters and GHGs. The length of an arrow represents the variance, and the cosine between two arrows represents the linear correlation between the two variables. All the variables are scaled to unit variance before performing PCA. The variables that are better explained by the two principal components will be longer and closer to the unit circle. Acute and obtuse angle represents positive and negative correlation, respectively, while a right angle implies a lack of correlation.

An anti-correlation between CH_4_ and wind speed (Fig. 5a) is found in monsoon. The wind rose diagram (Fig. 1d) also supports this finding. The prevailing south-westerly wind in monsoon is associated with low CH_4_ values in the wind rose diagram. A positive correlation between CO_2_ and wind speed is found. This interplay between CO_2_ and wind is discussed further in the following section. The association of CO_2_, CH_4_ with wind is reduced in post-monsoon (Fig. 5b). While a positive correlation between CO_2_ and CH_4_ is evident in the winter months (Fig. 5c). The correlation coefficient value between CO_2_ and CH_4_ in winter is 0.52 (n = 7108). The close association of CH_4_-PBL and CO_2_-soil temperature (both layers 1 and 2) is the dominant feature in pre-monsoon (Fig. 5d).

#### Influence of prevailing meteorology

Correlation coefficients (R) between wind speed and CO_2_ (R_CO2_) during monsoon, post-monsoon, winter and pre-monsoon are 0.51 (n = 118), 0.15, − 0.02 and − 0.28, while for CH_4_ are (R_CH4_) − 0.57 (n = 118), − 0.3, − 0.02 and − 0.27 respectively. A good inverse correlation between GHG and wind speed suggests that with an increase in wind speeds, GHG concentrations would decrease. In contrast, a weaker correlation would suggest regional/local transport plays some role^30,31^. Strong wind, especially during the monsoon season (Supplementary Fig. 1c) is likely to dilute the GHG concentration. This is validated for the case of CH_4_ in which the wind and CH_4_ concentration are negatively correlated (R_CH4_ = − 0.57). But, CO_2_ is positively related to wind speed (R_CO2_ = 0.51). To have further insight into the effect of wind on CO_2_, we take the average wind speed integrated over a larger area (up to 11.5° × 4.5°, covering the central to mid-Arabian sea) and re-calculate the correlation coefficient. The results (summarised below) show that in the case of CO_2_ the R-value practically remains the same, but for CH_4_ it is improved significantly.

- For area—(18–18.5° N) and (69.5–74° E) gives R_CO2_ = 0.53, R_CH4_ = (− 0.62)
- For area—(14–18.5° N) and (62.5–74° E) gives R_CO2_ = 0.54, R_CH4_ = (− 0.71)

A strong negative correlation between CH_4_ and wind presents dilution of CH_4_ due to intrusion of southern hemispheric clean air with a strong south-westerly wind of monsoon, schematically shown in Fig. 1a.

### Anthropogenic signature on GHG’s probability distribution

To investigate the anthropogenic and biospheric signature on GHGs, we have partitioned the CO_2_ and CH_4_ concentration for the day (07:00–18:00 LT) and night hours (20:00–06:00 LT) for the entire study period. Supplementary Fig. 4a,b shows the probability distribution (PD) of CH_4_ and CO_2_ concentrations during the daytime and nighttime, respectively. Supplementary Fig. 4b shows that the PD of CO_2_ is narrow (broad) during the night (day) time. Mean daytime and nighttime CO_2_ concentrations are 404.6 ± 7.8 ppm (µ ± 1σ) and 407.42 ± 5.93 ppm, respectively. On the other hand, the CH_4_ concentration in the daytime and nighttime are practically the same. The respective mean values are 1.974 ± 0.078 ppm and 1.968 ± 0.07 ppm. We have also calculated the skewness (S) and kurtosis (K) of these distributions. The lower skewness $\left( {{\text{S}}_{{{\text{CO}}_{{2}} }} \, = \,0.0{4}} \right)$ for nighttime distributions than that of the daytime distribution $\left( {{\text{S}}_{{{\text{CO}}_{{2}} }} \, = \,0.1{6}} \right)$ implies that the nighttime distribution is more symmetric. The same is the case for CH_4_, for which the values are 0.37 and 0.97, respectively. This means the nighttime distributions are more constrained. For CO_2_, the kurtosis values for both day (0.52) and nighttime (1.20) are much lower than those obtained for a normally distributed curve, which is 3. This may imply that the extreme values are less relative to the normally distributed curve, but compared to daytime, the nighttime emissions are characterised by a slightly more number of extreme values. However, CH_4_ shows the opposite behaviour, since the kurtosis value for daytime (2.7) is higher than that of the nighttime (− 0.24).

The probability distribution of CO_2_ and CH_4_ of day and nighttime data has also been carried out for different seasons. Supplementary Figs. 5a–d and 6a–d show the results. As found earlier, the monsoon season daytime PD is characterised by a broad peak, but the nighttime PD is relatively narrow. The nighttime mean (Supplementary Fig. 5a) is right-shifted, as there is practically no sink of CO_2_. The post-monsoon season shows a broader spectrum for both the period (Supplementary Fig. 5b), indicating an increase in the nighttime source of CO_2_. The PDs for the winter (DJF) and the pre-monsoon season (MAM) are quite broad, and they show similar characteristics (Supplementary Fig. 5c,d). Another feature of these distributions is the range of daytime CO_2_: the monsoon season has a range of 385–410 ppm, and the post-monsoon season 385–415 ppm. At the same time, the winter season shows a range of 402–425 ppm and the pre-monsoon season 405–435 ppm. Throughout the monsoon, the mean CO_2_ concentration is 400.22 ± 5.48 ppm during the day, whereas an elevated CO_2_ level, 406.57 (~ 6.35 ppm more than daytime) with low SD, is a vital feature of the nighttime variability (Table 1). This difference is also noticeable through the post-monsoon (ON), but the difference of mean day and night CO_2_ concentration gets decreased. During the winter and pre-monsoon (DJF and MAM) the difference during the day and night CO_2_ concentration almost vanishes.

**Table 1:** Season wise average concentration and standard deviation of GHGs during day and night.

	Monsoon		Post-monsson		Winter		Pre-monsoon	
	Day	Night	Day	Night	Day	Night	Day	Night
CO_2_	400.22 ± 5.48	406.57 ± 3.96	401.845 ± 6.27	404.05 ± 5.55	409.877 ± 4.51	409.56 ± 4.06	416.953 ± 5.43	417.01 ± 4.6
CH_4_	1.903 ± 0.0415	1.904 ± 0.0406	2.028 ± 0.0596	2.0204 ± 0.053	2.002 ± 0.0722	1.987 ± 0.0503	1.963 ± 0.0444	1.967 ± 0.0451

In comparison, CH_4_ does not show any significant daytime and nighttime variation in most seasons except winter. Figure 4b and Table 1 reveal a significant seasonal variation of CH_4_, but day–night variation in intra-seasonal timescale existed only in winter. Wintertime day and night mean CH_4_ concentration differs by 15 ppb. High mean CH_4_ in daytime indicates the source, and higher SD represents diversity in source processes of CH_4_ than night. This is also reflected in the S and K values of methane; the daytime values are high (S = 2.58, K = 11.33) for the winter season (DJF). Similarly, the pre-monsoon season (MAM) also shows relatively higher values (S = 2.28, K = 9.90). This means that methane concentrations in this region remain high from November through March due to enhanced emission and/or reduced loss due to the reduction in the OH radical^32^.

### Diurnal variation of CO_2_ and CH_4_

**Figure 6:**
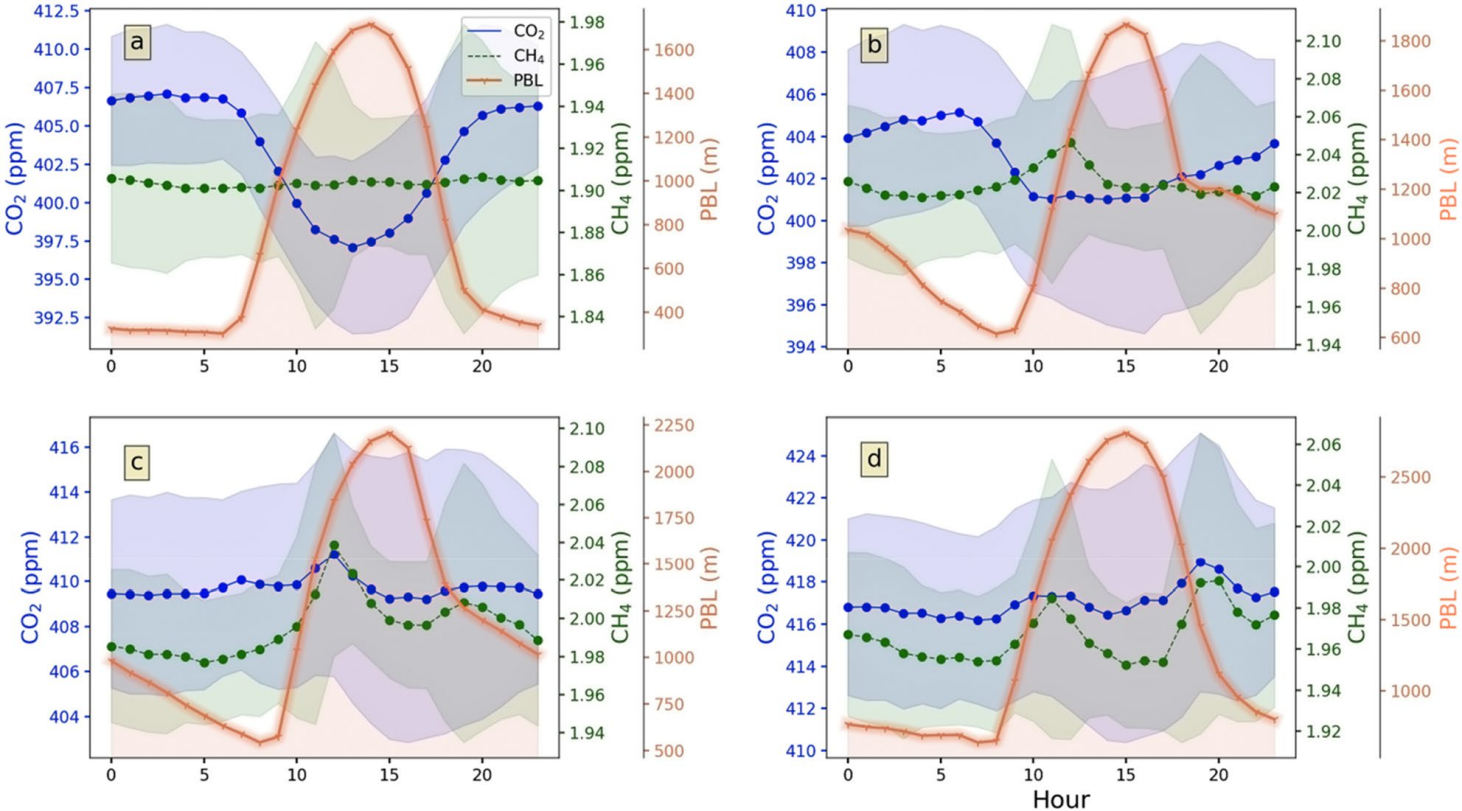
Diurnal variation of seasonal CO_2_, CH_4,_ and planetary boundary layer height (PBL) during (**a**) monsoon, (**b**) post-monsoon, (**c**) winter, and (**d**) pre-monsoon.

Figure 6a–d show the diurnal cycle of CO_2_ and CH_4_ over the *sng* site averaged over a seasonal cycle. During the monsoon season (Fig. 6a), the diurnal pattern of CO_2_ remains high in the early morning and then steadily decreases due to increased photosynthetic activity and becomes minimum around 13:00 LT. In the post-monsoon season (Fig. 6b), the minimum value is shifted to 10:00 LT. For the winter season and pre-monsoon (Fig. 6c,d), the patterns are very different; the maximum and minimum values are not well defined, and the diurnal pattern is somewhat linear. A large deviation from the monsoonal-pattern during the winter and pre-monsoon strongly indicates a weakening biospheric role and increased anthropogenic activities driving the diurnal behaviour of CO_2_ concentration in these seasons. On the other hand, the diurnal pattern of CH_4_ during the monsoon is not well defined. The patterns, however, are quite different for the other seasons, as illustrated in Fig. 6b–d; the minimum in the early hours and the maxima around 10:00 LT. The pre-monsoon season also gets second maxima around 19:00 LT.

Figure 6a–d shows the seasonal variation of the diurnally averaged Planetary Boundary Layer Height (PBLH) in association with CH_4_ and CO_2,_ respectively. Table 2 shows the amplitude, i.e., the difference between the diurnal minima and the maxima for different seasons. The table indicates that the diurnal variation of CO_2_ is low during the winter or pre-monsoon time (1.98 and 2.75 ppm, respectively). The variability is increased during post-monsoon (4.1 ppm) and obtains maximum amplitude (10.01 ppm) during the monsoon. Moreover, it is noted that PBL height attains its maximum value around 14:00–15:00 LT for almost every season while the time of lowest CO_2_ is different for different seasons. CO_2_ reaches a minimum of around 10:00 LT during post-monsoon (Fig. 6a,b), shifted to 12:00–13:00 LT during monsoon. This shifting may be related to the amount of vegetation around the site. Figure 4a suggests that NDVI (a proxy of vegetation) is high during October–November, which may lead to enhance photosynthesis during the noon hours (11:00–12:00 LT).

**Table 2:** Amplitude (maximum − minimum) of diurnal variation for the different seasons.

	JAS (monsoon)	ON (post-monsoon)	DJF (winter)	MAM (pre-monsoon)
CO_2_ (ppm)	10.01	4.133	1.98	2.75
CH_4_ (ppb)	5.3	28.79	62.05	40.96

Some interesting features are observed during the period 00:00–06:00 LT. CO_2_ levels remain somewhat constant for the monsoon and post-monsoon periods. Constant levels at night during monsoon and post-monsoon give evidence of continuous but weak sources such as plant and soil respiration. CH_4_ shows a maximum (minimum) diurnal amplitude (Table 2) of 62.05 (5.3) ppb during winter (monsoon). The monsoon to post-monsoon transition phase experiences the maximum increase in CH_4_ amplitude (around 443%). On the other hand, the pre-monsoon to monsoon transition registers a modest decrease (~ 87%) in CH_4_ diurnal amplitude.

## Discussion and conclusions

The seasonal amplitude of CO_2_, is high over *sng* as compared to *knp* (forest site), *amd* (urban site) and *sad* (semi-urban site) of India. The seasonality of *knp*-CO_2_ is mostly driven by the biosphere. Pre-monsoon rainfall in *knp* enhances Leaf Area Index (LAI), which in turn increases CO_2_ assimilation during daytime^11^ hence reducing the atmospheric CO_2_ concentration. While, during monsoon, though LAI is high, occasional overcast conditions reduce photosynthetically active radiation (PAR) from reaching the canopy, reducing the CO_2_ uptake. Simultaneously, *sad* shows enhanced CO_2_ concentration in pre-monsoon months due to higher temperature and solar radiation^26^ and minimum in monsoon mostly driven by enhanced photosynthesis with the availability of higher soil moisture. CO_2_ mixing ratio over *cri* is highest in February–March, due to increased heterotrophic respiration and anthropogenic activity in northern India^33^. The high seasonal amplitude of *sng* is characterised by low CO_2_ in monsoon and post-monsoon and elevated CO_2_ during pre-monsoon season. The steady growth of CO_2_ during the dry season (November to May) indicates a decreasing trend of vegetation uptake in the neighbouring regions (Fig. 4a). A sharp increase in mean value (410–417 ppm) during the pre-monsoon period could be attributed to enhanced solar radiation. Higher temperature enhances CO_2_ photosynthesis during daytime and respiration during the nighttime^34^. In that case, the diurnal amplitude (maximum–minimum) of CO_2_ should be high, but during pre-monsoon, this amplitude becomes negligible (discussed in diurnal variation of GHG section). Soil respiration and biomass burning also act as a source of CO_2_ into the atmosphere. With the advancement of monsoon season, the CO_2_ concentration steadily reduces mainly due to the CO_2_ uptake by the biosphere. Additionally, the reduction in temperature further decreases the leaf and soil respiration^35,36^. Moreover, NDVI (a proxy of vegetation) is increasing (Fig. 4a) during monsoon months.

CH_4_ concentrations over monsoon Asia (including China) show higher values during the wet seasons (JAS and ON) and low values during dry periods (DJF and MAM) driven by agricultural practices, i.e., paddy fields as well as large scale transport and chemistry^37,38^. Like the 'background' region, *mlo* in the Pacific Ocean*,* we have also observed low methane concentrations during the summer months (JAS, Fig. 2c) though the mechanism is not the same as that of *mlo*. In our case, low concentration is controlled by strong monsoon circulation though surface emission (from AW sector, Fig. 4b) is high. Low surface CH_4_ concentration instead of high local emission is also found by Guha et al.^39^. They suggest the intrusion of southern hemispheric clean air with monsoonal south-westerly wind is responsible for low surface CH_4_ concentration. Therefore, maximum CH_4_ concentration is found during post-monsoon when south-westerly current is decreased.

In comparison, the second maximum of CH_4_ emission is observed in February–March–April with very low NDVI. Hence, emission from wastewater and landfills, enteric fermentation, and animal waste management plays a dominant role in CH_4_ emission during February–March–April. It is found that boundary layer dynamics is not sufficient for the seasonal change of CO_2_ and CH_4_ levels. In a nutshell, the tropospheric CH_4_ concentration in this region is determined by the following processes: a balance between the local to regional scale surface emission, destruction by the OH radicals at the hemispheric scale, and the regional monsoon circulation. Meanwhile, a low concentration of CO_2_ instead of high positive residual flux (biosphere + fossil) indicates that monsoon flow brings cleaner air, which lowers the average concentration of atmospheric CO_2_ over *sng* as observed for CH_4_. Hence, we found a strong negative correlation between wind speed and CH_4_. But interestingly, a positive correlation is evident between CO_2_ and wind speed in monsoon.

Monsoon rainfall frequently comprises wet and dry spells of precipitation over a period of 10–90 days, widely known as monsoon intraseasonal oscillation (ISO). 10–20 days^40^ and 20–60 days^41,42^ are two dominant modes of ISO. Cross equatorial low-level jet (LLJ, surface south-westerly wind) is a dominant feature of monsoon. LLJ also shows intraseasonal oscillation in association with monsoon ISO^43^ or precisely with north/north-eastward propagation of deep convection^44^, but with a lag of about 2–3 days. Valsala et al.^45^ also show the interplay between monsoon ISO and net biosphere CO_2_ flux. OLR is considered a proxy for the deep convection and is used for precipitation estimation^46–48^. A lag correlation analysis is carried out between filtered (10–60 days band passed) wind vs. filtered OLR and filtered CO_2_ vs. filtered wind (see “Supplementary section”). A maximum correlation is observed between OLR and wind when OLR leads the wind by 2–3 days.

In contrast, CO_2_ shows a strong positive correlation with the wind, with wind lagging 1–2 days. Hence, the positive correlation between CO_2_ and wind may arise due to the response of monsoon intraseasonal oscillation. A strong monsoon circulation brings cleaner air, which reduces the CO_2_ and CH_4_ both, but CO_2_ is modulated by biospheric uptake. The biosphere uptake is further modulated by monsoon intraseasonal oscillation. Consequently, we found a positive relation between CO_2_ and wind as a response to monsoon.

A higher SD of CO_2_ histogram during the daytime indicates a broader spread with respect to the nighttime distribution, which is characterised by a lower SD. So, the broadness of the CO_2_ distribution function during the daytime is caused by a diverse source/sink of CO_2_. With the development of the boundary layer, CO_2_ gets mixed vertically. As the day progress, the photosynthetic CO_2_ sink reduces the CO_2_ concentration, which is moderated by the increase in PBLH. While anthropogenic sources of CO_2_ and plant respiration are also active during the day, a broader CO_2_ distribution spectrum is yielded during different hours of the day. The narrow PD for CO_2_ in the nighttime is suggesting the dominating role of CO_2_ release by respiration and anthropogenic activity. The difference between daytime and nighttime CO_2_ distribution is evident in monsoon and post-monsoon only. Moreover, the diurnal variation of CO_2_ is also most prominent in these seasons. Daytime CO_2_ minima (around noon), a constant value of CO_2_ during the night (00:00–06:00 LT), different daytime and nighttime CO_2_ histogram are the key features in monsoon and post-monsoon season. In contrast, the diurnal variation of CO_2_ in winter and pre-monsoon diminishes. Though, daytime PBLH maximum is more (> 2000 and > 2500 m) during winter and pre-monsoon (Fig. 6c,d), which indicates a strong mixing. This clearly shows that the monsoon and post-monsoon season get their CO_2_ share mainly from the active biosphere. In contrast, the other two seasons get from the degradation of the biosphere and anthropogenic activities. Boundary layer dynamics are ineffective when vegetation is less. Moreover, the close association of soil temperature at level1 and 2 with CO_2_ (Fig. 5d) implies that soil respiration is a dominant part of pre-monsoon CO_2_.

A similar pattern in CH_4_ histogram during daytime and night time implies that the source and transport processes of CH_4_ remain more or less invariant (note that the CH_4_ sink by OH is a slow process, with a time scale of 1 year or longer in summer over the tropics; Patra et al.^17^). Diurnal variation of CH_4_ (Fig. 6a–d) shows morning CH_4_ develops with the advent of PBLH other than monsoon. Such a pattern suggests, trapped CH_4_ in the neighbouring valley due to a stable boundary layer of the previous night becomes available at our site (top of a hill) with the rise in the boundary layer in the morning hours. So, we get a morning peak in CH_4_ concentration. As PBLH grows beyond the site elevation, CH_4_ drops due to mixing with a larger area. Winter is characterised by a small peak in CH_4_ levels (Fig. 6c) at the evening (around 19:00 LT), which further develops and emerges as a dominant peak in pre-monsoon. Hence, a close association of PBL and CH_4_ is observed in biplot in pre-monsoon (Fig. 5d).

### Data and methodology

#### Climatology of the study area

The mean monthly variation of relative humidity (RH in %) and temperature (°C) from NCEP-FNL reanalysis dataset over *sng* is shown in Supplementary Fig. 1a,b during the period 2014–2015. Temperature over *sng* varies from ~ 25 to ~ 31 °C. Relative humidity (RH) was maximum during south-west monsoon (June-July–August–September, JJAS) season of > 75%, and the minimum occurred during winter (December–January–February, DJF) of about < 50%. At *sng*, the wind speed at 850 hPa (data source: ERA-Interim) varies between 1 and 12 ms^−1^. Maximum wind speed occurred mainly from the south-west direction during the Indian summer monsoon (ISM) months, JJAS, which originated from the Arabian Sea. In winter, the winds are mostly from the northeast direction, originated from the Indian subcontinent (Supplementary Fig. 1c,d). Figure 1b shows the location of the study area with the mean outgoing longwave radiation (shaded) and mean wind (1000 hPa) flow in vector form. Figure 1a depicts the south-westerly monsoon flow from the ocean to land with enhancing convection (low OLR) over the Indian sub-continent. Figure 1b illustrates an opposite flow pattern during the winter associated with suppressing convection (high OLR). So, it is evident that our study area experiences a strong seasonally reversing of the wind flow from summer to winter. The wind rose diagram shows south-westerly wind is associated with low CO_2_ and CH_4_ concentration (Fig. 1c,d). The interplay between wind and GHG concentration is discussed further in “Influence of prevailing meteorology” section.

### GHG analyser

Continuous air sampling was done through a fast greenhouse gas analyser (model: LGR-FGGA-24r-EP) from a 10 m meteorological tower. It is based on enhanced off-axis integrated cavity output spectroscopy (OA-ICOS) technology^49^. This instrument is able to provide CH_4_, CO_2,_ and H_2_O concentration simultaneously with high temporal resolution (up to 1 Hz). The sensor was calibrated using a zero air cylinder having known CO_2_, CH_4_ concentrations. The 'dry values' of CO_2_ and CH_4_ mixing ratios, corrected for water vapour, are reported in this paper. The CO_2_ and CH_4_ data integrated for 100-s intervals are presented here. The analyser has 0.3 ppb, 0.05 ppm, and 5 ppm precision of CH_4_, CO_2,_ and H_2_O when operating in the 0.01 Hz frequency. Moreover, we take 15-min average CO_2_ and CH_4_ measurements for further analysis. The site has been operational from July-2014 to November-2015. There were several data gaps in between, with an opening from 3-May-2015 to 9-July 2015 (longest gap), due to instrument maintenance. This gap is filled with weekly flask samples data^24^ obtained from the same site. CO_2_ and CH_4_ concentration data have been plotted on diurnal and monthly time scales. The year was divided into four different seasons, i.e., monsoon (July–August–September), post-monsoon (October–November), winter (December–January–February), and pre-monsoon (March–April–May).

Due to the unavailability of AWS in the study area, no in-situ meteorological data were available; instead, we use different kinds of reanalysis data as mentioned later.

### Kaziranga (***knp***) CO_2_ data

The Metflux India flux observational site Kaziranga National Park (*knp*) is a semi-evergreen forest located in the north-eastern state of Assam. The CO_2_ concentration over the forest is measured at the height of 37 m using an enclosed path CO_2_–H_2_O infrared gas analyser (LI-7200, LI-COR, USA) at frequency of 10 Hz. The high-frequency data are processed using the EddyPro software and averaged in the time interval of 15 min. The details of the study area and instruments can be found in^11^.

### Moderate-resolution imaging spectrometer (MODIS)

The MODIS was launched in December 1999 on the polar-orbiting NASA-EOS Terra platform^50,51^. It has 36 spectral channels covering visible, near-infrared, shortwave infrared, and thermal infrared bands. In the present study, we have used 5-km spatial resolution having 16-day temporal resolution NDVI (Normalized difference vegetation index) data. We got the dataset from MODIS (Product-MOD13C1) official website ("https://modis.gsfc.nasa.gov/data/dataprod/mod13.php"). The NDVI is a normalised transform of the near-infrared (NIR) to red reflectance ratio (RED) and calculated using the following equation

$$NDVI = \frac{NIR - RED}{{NIR + RED}}$$

NDVI values range from − 1.0 to + 1.0. Higher positive values are associated with increased vegetation coverage. The NDVI is averaged over the region 18–18.5° N and 73.5–74° E.

### Outgoing longwave radiation

Outgoing longwave radiation (OLR) is the radiative flux leaving the earth-atmosphere in the infrared region. OLR has a broad wavelength ranging from 4 to 100 µm. In the present study, we have been using OLR data from a very high-resolution radiometer (VHRR), onboard Kalpana-1 satellite. VHRR measures OLR in infrared (10.5–12.5 µm) and water vapour (5.7–7.1 µm) wavelength band. Retrieval algorithm of OLR from the VHRR images, archived at the National Satellite Data Centre of the India Meteorological Department, New Delhi, is available in^52^. The OLR data is available at three-hour intervals (i.e. 00, 03, …, 18 and 21 UTC) starting from May-2004 over the Indian region (40° S–40° N, 25° E–125° E). It has 0.25° × 0.25° spatial resolution. In the present study, we used daily data. The yearly data files are available on the official site of IITM, Pune ("https://www.tropmet.res.in/~mahakur/Public_Data/index.php?dir=K1OLR/DlyAvg"). Usually, low OLR values (< 200 W m^−2^) denote convection, whereas high values indicate clear sky. OLR is averaged over the region 18.12–18.62° N and 73.62–74.12° E.

### Modern-era retrospective analysis for research and applications (MERRA)

The MERRA-2 is a NASA atmospheric reanalysis project that began in 1980. It replaced the original MERRA^53^ reanalysis product using an upgraded version of the Goddard Earth Observing System Model, Version 5 (GEOS-5) data assimilation system. MERRA-2 includes updates to the model^54^ and Global Statistical Interpolation (GSI) analysis scheme of Wu et al.^55^. MERRA-2 has a spatial resolution of 0.625° × 0.5°. In the present study, we used MERRA-2 dataset to determine the Planetary Boundary Layer Height on an hourly timescale. We take planetary boundary later height (PBLH) averaged over 18–18.5° N and 73.13–74.38° E for our study region.

### European re-analysis-interim (ERA-Interim)

Era-Interim is a reanalysis product of the global atmosphere produced by the European Centre for Medium-Range Weather Forecast (ECMWF) available from 1979^56^. The Era-Interim atmospheric model and reanalysis system uses cycle 31r2 of ECMWF’s Integrated Forecast System (IFS). The system includes 4-dimensional variational analysis (4D-Var) with a 12-h analysis window. In each window, available observations are combined with prior information from a forecast model to estimate the evolving state of the global atmosphere and its underlying surface. Meridional and zonal wind components at 850 hPa at a spatial resolution of 0.25° × 0.25° (grid dimension: 18–18.5° N and 73.5–74° E) were used.

### ERA5

ERA5 is the latest version of reanalysis produced by ECMWF. ERA5 is produced using 4D-Var data assimilation in ECMWF's Integrated Forecast System. A temporal resolution of 1 h and a vertical resolution of 137 hybrid sigma model levels. The 37 pressure levels of ERA5 are identical to ERA-Interim^57^. ERA5 assimilates improved input data that better reflects observed changes in climate forcing and many new or reprocessed observations that were not available during the production of ERA-Interim.

ERA5-Land provides the land component of the model without coupling to the atmospheric models. It uses the Tiled ECMWF Scheme for Surface Exchanges over Land with revised land-surface hydrology (HTESSEL, CY45R1). It is delivered at the same temporal resolution as ERA5 and with a higher spatial resolution of 0.1° × 0.1°. 2 m air temperature, soil temperature level 1 (0–7 cm), and soil temperature at level 2 (7–28 cm) is used.

### NCEP FNL re-analysis

The NCEP FNL (final) operational global analysis data are on 1° × 1° grid prepared operationally every six hour. This product comes from the Global Data Assimilation System, which continually gathers observational data. The time series of the archive is continually extended to a near-current date but not preserved in real-time (http://rda.ucar.edu/datasets/ds083.2/). The key aim of these re-analysis data is to provide compatible, high-resolution, and high-quality historical global atmospheric datasets for use in weather research communities^58, 59^. Air temperature and RH are averaged over the area 18–19° N and 73–74° E.

## Supplementary Information

Supplementary Information.
