# Supplementary material for: Diurnal and seasonal variability of CO2 and CH4 concentration in a semi-urban environment of western India
Source: Sci Rep. 2021 Feb 3;11:2931. doi: 10.1038/s41598-021-82321-1 (PMC7859198; doi:10.1038/s41598-021-82321-1)
Supplement: Supplementary file 1 — Supplementary Information. [file 41598_2021_82321_MOESM1_ESM.pdf]

Diurnal and seasonal variability of CO_2 and CH_4 concentrations in a semi-urban environment of western India

Abirlal Metya[a,b], Amey Datye[a], S. Chakraborty[a,b*], Yogesh K. Tiwari[a], Dipankar Sarma[a], Abhijit Bora[c], Nirmali Gogoi[c]

[a] *Indian Institute of Tropical Meteorology, MoES, Pune, 411008, India*

[b] *Department of Atmospheric and Space Sciences, Savitribai Phule Pune University, Pune, 411007, India*

[c] *Department of Environmental Science, Tezpur Central University, Tezpur, India*

Supplementary Figure:

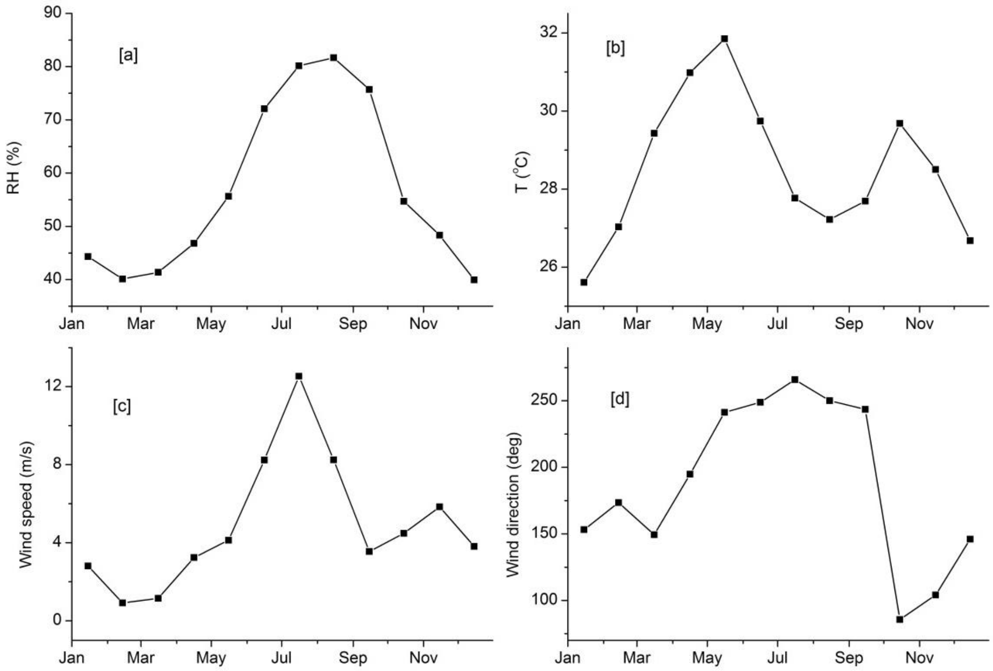

Supplementary Figure 1: Seasonal variation of a) Relative Humidity b) Surface Temperature c) Wind speed (at 850hPa) and d) Wind direction averaged during 2014–15. The datasets were retrieved from the NCEP-NCAR reanalysis data.

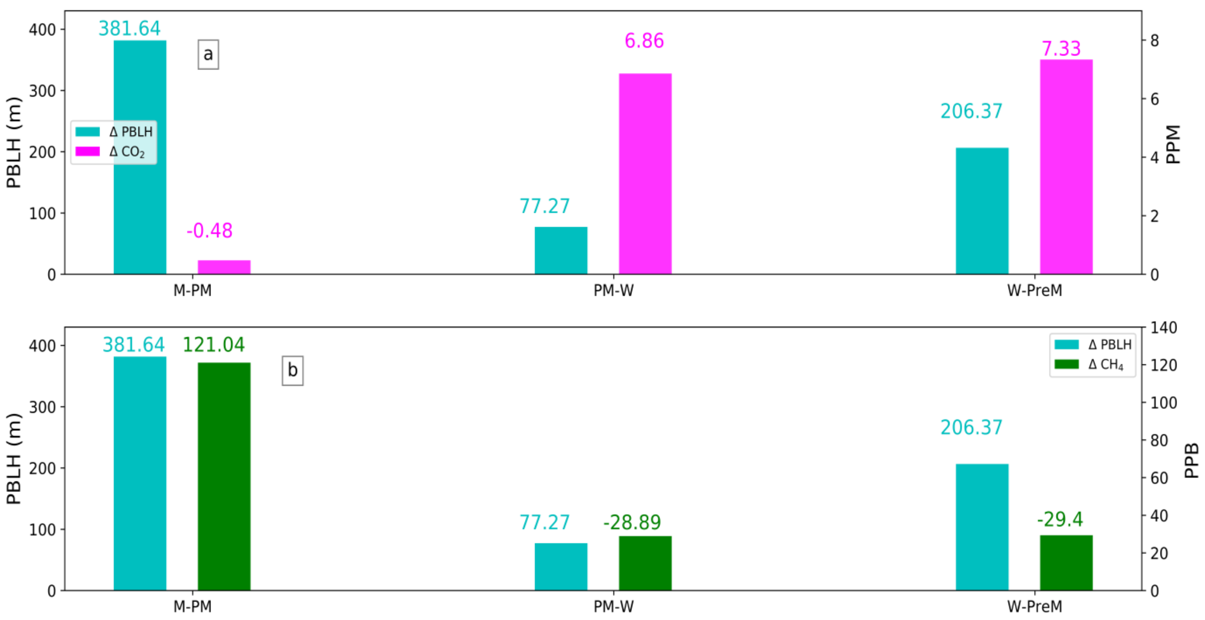

Supplementary Figure 2: Seasonal differences in BLH against respective change in (a) CO_2 and (b) CH_4. Cyan coloured bar presents ΔPBLH, magenta and green bar presents ΔCO_2 and ΔCH_4 respectively.

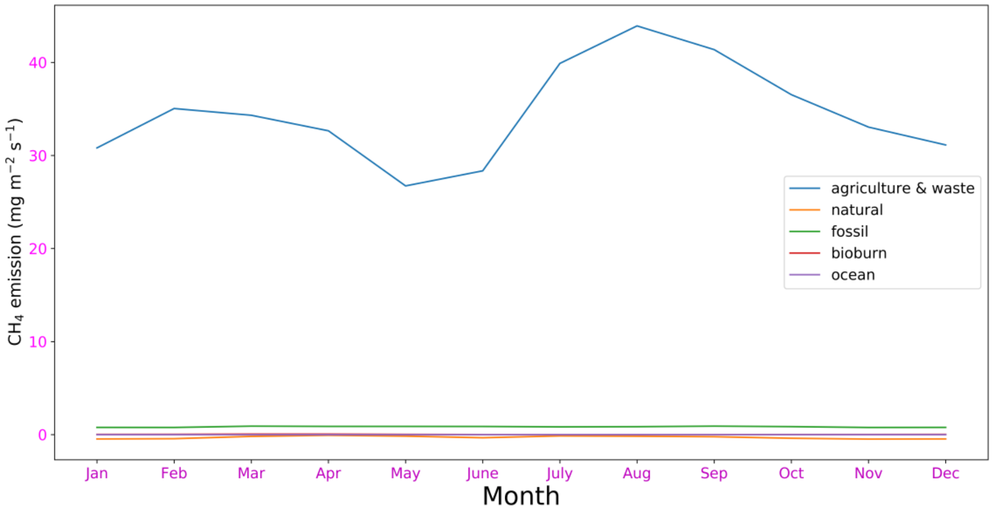

Supplementary Figure 3: Sector-wise CH_4 emission from Carbon Tracker (CT).

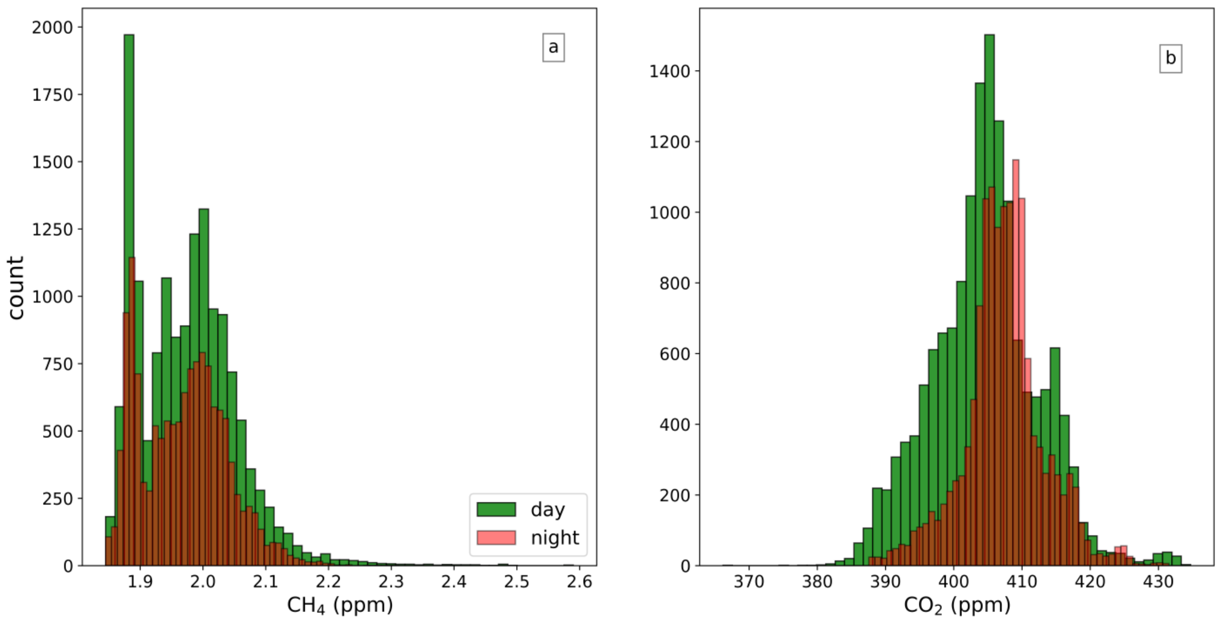

Supplementary Figure 4: Probability Distribution of (a) CH_4 and (b) CO_2 during daytime (green bars) and night-time (orange bars).

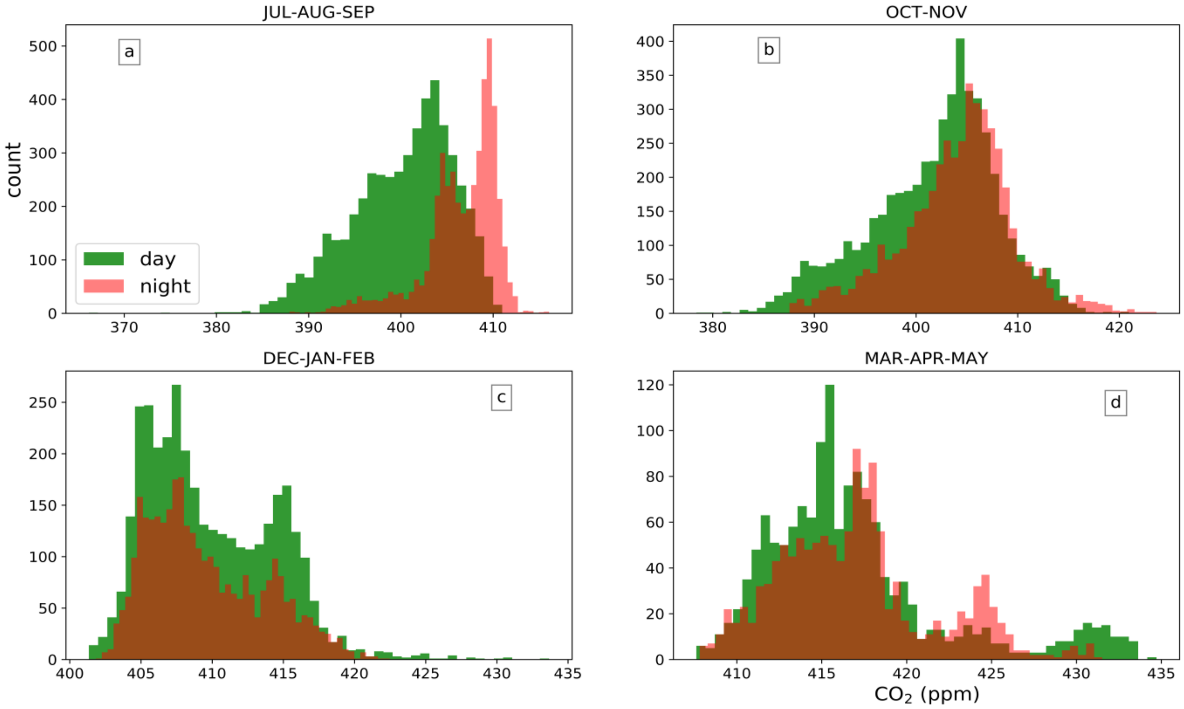

Supplementary Figure 5: Probability Distribution of CO_2 during day time and night time for (a) Monsoon, (b) Post-Monsoon, (c) Winter and (d) Pre-Monsoon.

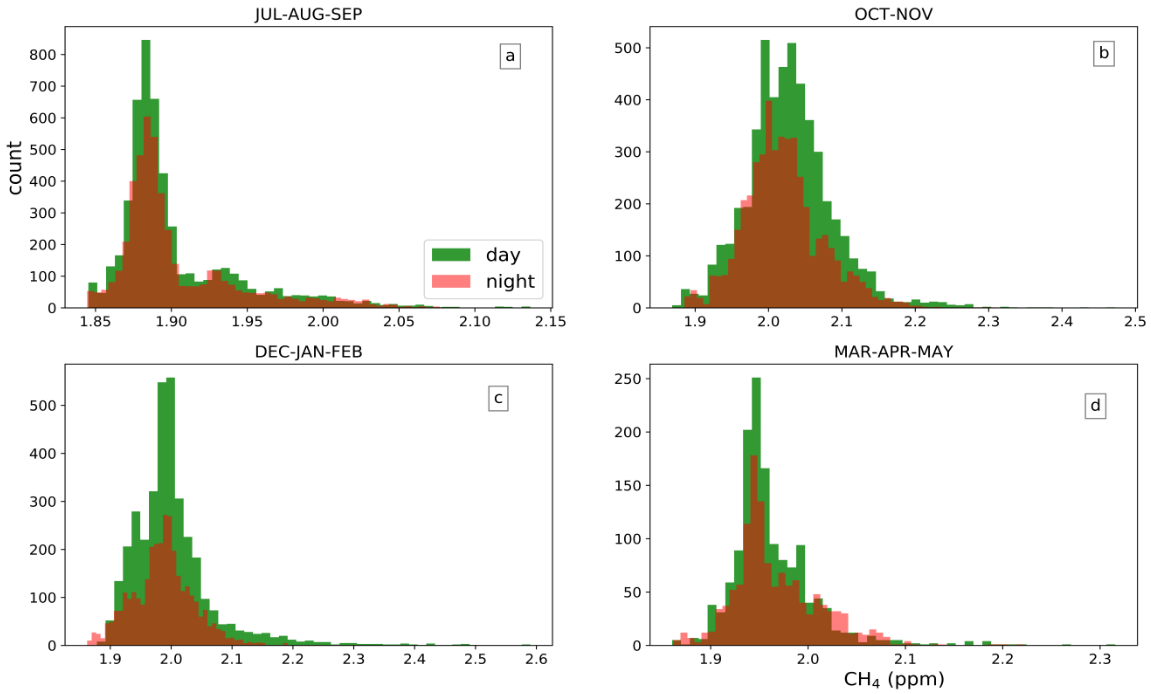

Supplementary Figure 6: Probability Distribution of CH_4 during day time and night time for (a) Monsoon, (b) Post-Monsoon, (c) Winter and (d) Pre-Monsoon.

1. Data used:

1.1. CarbonTracker (CT):

We use CO_2 concentration and terrestrial biospheric CO_2 flux from CarbonTracker version 2019 (CT2019), which is developed and maintained by the Earth System Research Laboratory (ESRL) at National Oceanographic and Atmospheric Administration (NOAA) (Peters et al., 2007). This data set provides optimized terrestrial biosphere CO_2 flux and CO_2 concentration at a spatial resolution of 1° X 1° horizontal grids and at a temporal resolution of three hourly time steps from the year 2000 to 2018. Daily-mean data of CO_2 concentration and biosphere CO_2 flux during monsoon (JJAS) of 2015 is used in this study.

CarbonTracker-CH_4 [1] is a data assimilation system that optimizes global CH_4 fluxes. It is based on an ensemble Kalman smoother with a fixed lag assimilation window. CarbonTracker-CH_4 provides CH_4 flux from several sectors in a monthly time scale with 1° X 1° horizontal resolution.

1.2. Lead-lag relation between CO_2, OLR, and Wind:

Lead-lag correlation analysis is carried-out between wind vs. OLR, CO_2 vs. OLR, and CO_2 vs. wind. OLR is taken from IITM-OLR product and averaged over the area (18-20° N and 73-76°E). The wind is taken from Era-Interim and averaged over the area (10-20°N and 62.5-67.5°E). The first three harmonics are removed from wind and OLR using a Fourier filter. Then a 10-60 day band-pass filter is applied. CO_2 and CH_4 time series have more missing values in 2014-monsoon than 2015-monsoon. Hence, all the lead-lag correlations are carried out on 2015. A multiple linear regression equation is fitted to predict the missing values of CO_2 –

$$CO_2 = a_1[PBLH] + b_1[CO_2]_{CT} + c_1\left[CO_{2_{bio}}\right]_{CT} + d_1[u] + e_1[v] + f \qquad (1)$$

Where [PBLH] is the daily planetary boundary layer height (in meter) from MERRA , $[CO_2]_{CT}$ is the CO_2 concentration (in ppm) obtained from CT2019, $\left[CO_{2_{bio}}\right]_{CT}$ terrestrial biosphere CO_2 flux (in $gCm^{-2}s^{-1}$) from CT2019 over the region- 18°-20°N and 73°-76°E.

[u] and [v] are the x and y component of wind (in ms^{-1}) obtained from Era-interim over the region (20°-10°N and 62.5°-67.5°E) and a_1, b_1, c_1, d_1 and e_1 are the coefficients, and f is the constant term.

After finding the best fit, the equation becomes

$$CO_2 = -0.59[PBLH] - 3.16[CO_2]_{CT} + 0.99\left[CO_{2_{bio}}\right]_{CT} + 4.08[u] - 1.21[v] + 404.92 \qquad (2)$$

The correlation coefficient between predicted and observed CO_2 is 0.82 (n=60 , p =0.01). We have predicted CO_2 concentration for the period 1-June-2015 to 30-September-2015 using equation 2. Then a band-pass filter (10-60 days) is applied to the predicted CO_2 concentration.

A lead-lag correlation analysis is carried out between filtered wind and OLR (Supplementary Figure 7a) time series. It shows a good association of wind and OLR, with OLR is leading by 2-3 days. A similar result is also found in Joseph and Sijikumar[2]. They suggest intensification (weakening) of LLJ over the Arabian Sea during monsoon active (break) spells with a certain lag. A second lead-lag correlation analysis is also carried out between CO_2 and OLR (Supplementary Figure 7b), where OLR is leading by 1-day. Valsala et al.[3] found intraseasonal oscillation of monsoon rainfall and biosphere CO_2 flux showing a coherent structure with 2-3 days lag (CO_2 flux lags rainfall). Hence, the lag relation between CO_2 concentration and OLR (Supplementary Figure 7b) can be attributed to the responses of the biosphere to the intraseasonal oscillation of monsoon, which affects atmospheric CO_2 concentration. Supplementary Figure 7c shows a maximum correlation between CO_2

concentration and wind at 1-day lag, with wind lagging behind CO_2. Hence, the coherence of CO_2 and wind can be attributed to the response of monsoon intraseasonal oscillation.

The major caveat of this analysis is that CO_2 concentration data is not continuous, having gaps in between. So, we have to simulate CO_2 concentration using a multiple linear regression using LLJ as an input variable. Moreover, predicted CO_2 captures (r^2=) 67% of the variability of observed CO_2. Hence, the maximum correlation of CO_2 and wind may be showing higher values than actual.

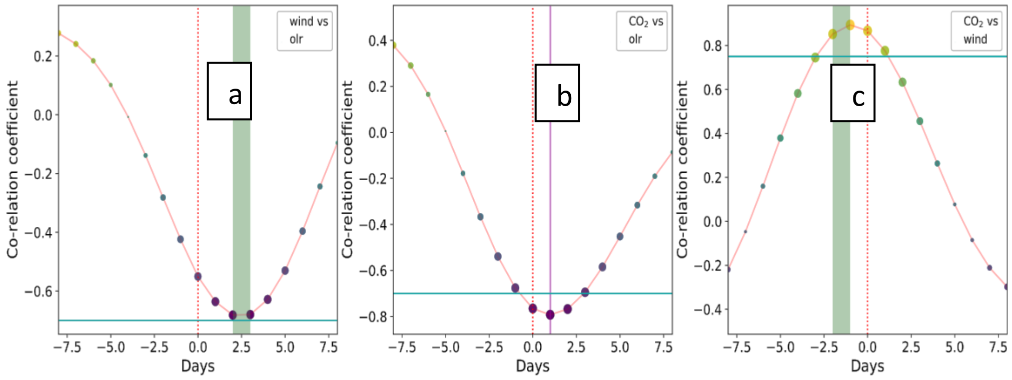

Supplementary Figure 7: Lead-lag correlation between (a) wind and OLR (b) CO_2 and OLR (c) CO_2 and wind. In the legend box, the second parameter is leading or lagging. For example: in (a) OLR is leading the wind.

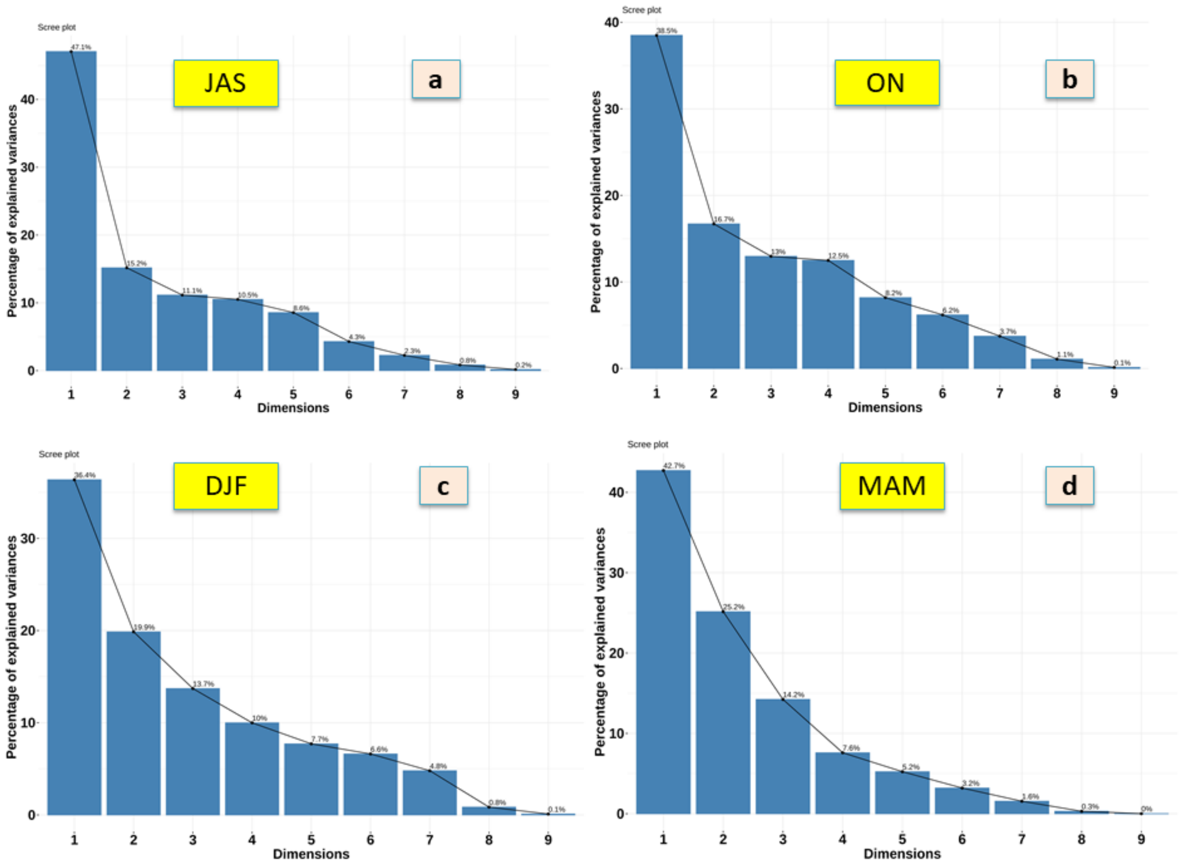

Supplementary Figure 8: *Scree Plot during (a) monsoon (JAS); (b) post-monsoon (ON); (c) winter (DJF) and (d) pre-monsoon (MAM). x-axis shows the principle components, i.e., '1' refers to PC1, '2' refers to PC2 etc.*

2. References

1. Peters, W. *et al.* An atmospheric perspective on North American carbon dioxide exchange: CarbonTracker. *PNAS* **104**, 18925–18930 (2007).
2. Joseph, P. V. & Sijikumar, S. Intraseasonal Variability of the Low-Level Jet Stream of the Asian Summer Monsoon. *J. Climate* **17**, 1449–1458 (2004).
3. Valsala, V. *et al.* Intraseasonal variability of terrestrial biospheric CO2 fluxes over India during summer monsoons. *Journal of Geophysical Research: Biogeosciences* **118**, 752–769 (2013).
